# A novel language-neutral Visual Cognitive Assessment Test (VCAT): validation in four Southeast Asian countries

**DOI:** 10.1186/s13195-017-0333-z

**Published:** 2018-01-22

**Authors:** Levinia Lim, Tze Pin Ng, Anam Paulus Ong, Maw Pin Tan, Alvin Rae Cenina, Qi Gao, Adeline Ng, Nagaendran Kandiah

**Affiliations:** 1 Department of Neurology, National Neuroscience Institute, Singapore, Singapore; 2 Gerontology Research Programme, Department of Psychological Medicine, National University Health System, Yong Loo Lin School of Medicine National University of Singapore, Singapore, Singapore; 3 Faculty of Medicine, Department of Neurology, Hasan Sadikin Hospital, Padjadjaran University, Kota Bandung, Jawa Barat Indonesia; 4 Department of Medicine, Faculty of Medicine, University of Malaya, Jalan Universiti, Kuala Lumpur, Malaysia; 5 Department of Neurosciences, Philippine General Hospital, University of the Philippines, Manila, Philippines; 6 Department of Neurosciences, Asian Hospital and Medical Center, Manila, Philippines; 7 Duke-NUS, Graduate Medical School, Singapore, Singapore

**Keywords:** Dementia, Mild cognitive impairment, Alzheimer’s disease, Cognitive test, Screening tool

## Abstract

**Background:**

Cognitive screeners are imperative for early diagnosis of dementia. The Visual Cognitive Assessment Test (VCAT) is a language-neutral, visual-based test which has proven useful for a multilingual population in a single-center study. However, its performance utility is unknown in a wider and more diverse Southeast Asian cohort.

**Methods:**

We recruited 164 healthy controls (HC) and 120 cognitively impaired (CI) subjects- 47 mild cognitive impairment (MCI) and 73 mild Alzheimer’s disease (AD) dementia participants, from four countries between January 2015 and August 2016 to determine the usefulness of a single version of the VCAT, without translation or adaptation, in a multinational, multilingual population. The VCAT was administered along with established cognitive evaluation.

**Results:**

The VCAT, without local translation or adaptation, was effective in discriminating between HC and CI subjects (MCI and mild AD dementia). Mean (SD) VCAT scores for HC and CI subjects were 22.48 (3.50) and 14.17 (5.05) respectively. Areas under the curve for Montreal Cognitive Assessment (0.916, 95% CI 0.884–0.948) and the VCAT (0.905, 95% CI 0.870–0.940) in discriminating between HCs and CIs were comparable. The multiple languages used to administer VCAT in four countries did not significantly influence test scores.

**Conclusions:**

The VCAT without the need for language translation or cultural adaptation showed satisfactory discriminative ability and was effective in a multinational, multilingual Southeast Asian population.

## Background

Dementia prevalence is estimated to rise considerably across Asia [1–3], as the elderly population is projected to increase from the current 10% to 24% of the total Asian population by 2050 [4–7]. This significant increase in prevalence of dementia is of immediate medical and social concern for Asian countries since cognitive decline is often associated with loss of independent function [8], loss of employment, and additional burden to family members and the healthcare system [9–11]. The economic burden of dementia, estimated at US$73 billion annually [12], is a major public health concern, especially for developing Asian countries. In the face of this dementia epidemic, it is crucial that clinicians are equipped with appropriate cognitive screening tools that can effectively detect dementia at an early stage [13, 14]. Early detection would allow interventions to retard the progression of dementia, more time for individuals and families to cope with this devastating illness, and a window for policy-makers to allocate much-needed resources.

Cognitive screening tools have proven to be simple, useful, and efficient in detecting early cognitive impairment [15, 16]. However, the diversity of languages across the world poses a significant barrier in using cognitive screening tools in an effective and efficient manner. In Asia, relatively lower education, lack of a common regional language, and existence of numerous dialects pose a major obstacle in using cognitive screening tools for the early diagnosis of dementia. Languages spoken in Asia fall into many differing language families, including Indo-European, Sino-Tibetian, and Austronesian with varying writing systems [17]. Based on the writing system classification of languages, Malay which is widely used in Malaysia, Indonesia, and Singapore as well as Tagalog which is widely used in the Philippines belong to the alphabetic group. On the other hand, Mandarin widely used in Singapore, Malaysia, and Indonesia belongs to the logographic group [18]. Thus when cognitive screening tools which were originally developed using English, an alphabetic language, are translated to logographic language, often new cognitive test items are required to replace items that cannot be translated. As a result, no universal screening tool can cater to the vast variety of languages spoken throughout Asia. While most cognitive screeners were designed for English language speakers, translation and adaptation of these tools into Asian languages, although useful, often result in alteration of their original neuropsychological and psychometric constructs. There is evidence to demonstrate that when using cognitive screening tools that have been modified or translated to meet local demands, these tests often result in overdiagnosis of cognitive impairment in non-English speakers [19]. Furthermore, the lack of standardized cognitive screening tools across Asian countries [20] will prevent meaningful cross-cultural comparisons and poses major challenges when conducting international clinical trials with cognition as outcome measures [21].

The Visual Cognitive Assessment Test (VCAT) is a visual-based cognitive screening tool designed to detect early cognitive impairment [22]. It is language neutral and encourages simple application to multilingual populations without the need for translation of test content. The VCAT is a 30-point test that evaluates memory, executive function, visuospatial function, attention, and semantic knowledge. The test items for each cognitive domain are visual based, with pictures and figures selected from the International Picture Naming Project and locally validated in older adults. Pictures that were easily and accurately identified by the participants were used to develop test items. The memory domain of the VCAT includes immediate and delayed recall of a visual scenario as well as recall of shapes and objects, while the executive function items require patients to figure out the mechanisms of gear rotation, recognizing patterns, and categorization of pictures. The language domain contains an item on category fluency and one on naming of pictures. The visuospatial items test patients’ ability to perform spatial reconstruction and grid navigation. Lastly, the attention domain consists of a shape cancellation task. The VCAT was recently demonstrated to be useful in a multilingual population in a single-center study [22], where the test’s diagnostic and discriminative validities were compared against the Mini Mental State Examination (MMSE) and Montreal Cognitive Assessment (MoCA). Its performance superseded MMSE in detecting early cognitive impairment and was comparable to MoCA with the added advantage of just having a single version of the test as there is no need to perform translation or adaptation. The area under the curve (AUC) of the VCAT for detection of cognitive impairment was 93.3 (95% CI 90.1–96.4). The sensitivity and specificity of the VCAT for diagnosis of cognitive impairment (MCI and mild AD) were 85.6% and 81.1% respectively.

In this study we evaluated the performance of the VCAT in a multinational, multicenter study in four linguistically diverse Southeast Asia populations and investigated the influence of different language families and writing systems on test performance across healthy controls (HC) and patients with cognitive impairment (CI).

## Methods

### Participants

This prospective, multicenter study was carried out across Singapore, Malaysia, Indonesia, and the Philippines. A total of 284 participants were recruited between January 2015 and August 2016. In all, 138 participants were recruited in Singapore from the National Neuroscience Institute Specialist Outpatient Memory Clinic and the Singapore Longitudinal Aging Study, 67 participants were recruited from the memory clinic of Hasan Sadikin Hospital in Indonesia, 40 participants were recruited from the Division of Geriatric Medicine, University of Malaya in Malaysia, and 39 participants were recruited from Asian Hospital & Medical Center and Manila East Medical Center in the Philippines. Inclusion criteria included subjects with mild dementia of the Alzheimer’s disease (AD) type, mild cognitive impairment (MCI), and healthy controls (HC). Diagnosis of dementia was based on the DSM-IV TR criteria [23], and AD was based on the NIA-AA criteria [24]. To ensure that only patients with mild dementia are recruited, a Clinical Dementia Rating (CDR) [25] score of 1 was required. MCI was diagnosed based on Petersen’s criteria [26]. Subjects were required to have symptoms in one or more cognitive domains, remain independent in all instrumental activities of daily living, a MMSE score > 24, and a CDR score of 0.5. HC were required to have no cognitive symptoms, be independent on all instrumental activities of daily living, a MMSE score > 27, and a CDR of 0. Only participants aged 50 years and older with at least 6 years of education were included in the study. From our previous experience, 6 years of education has been shown to be the minimum education required for subjects to be able to complete all of the cognitive assessments required for this study. Subjects with a Geriatric Depression Scale (GDS) score of 10 or more, suggestive of major depression, were also excluded. Participants were then classified into either the HC group or the CI group which consists of both the MCI and mild AD subjects.

All participants were tested in a private and quiet environment by trained raters. Ethical approval was obtained from the Centralized Institutional Review Board in Singapore, University Malaya Medical Center Ethics Committee in Malaysia, Bandung Adventist Hospital Institutional Review Board in Indonesia, and Asian Hospital Institutional Review Board in the Philippines. Informed consent was obtained from all participants and all methods were performed in accordance with the journal’s guidelines and regulations.

### Cognitive assessments

During each interview, the participant’s basic demographic information, including age, gender, race, years of education, and employment status, were collected. The MMSE [27], MoCA [28], VCAT [22], and GDS [29] were administered to each participant. The MMSE, MoCA, and VCAT were performed to assess global cognitive performance, which includes memory, language, executive function, visuospatial abilities, and attention. Locally validated and translated versions of the MMSE and MoCA were used in respective countries while a single version of the VCAT (without translation or modification) was used across all countries. These three scales have a score range from 0 to 30 points where lower scores indicate greater cognitive impairment. In order to ensure the uniformity of administration and accuracy in scoring of the newly introduced VCAT, all local (Singapore) raters were trained face to face while the overseas raters were trained through video conferencing. From our previous work, we used a VCAT cutoff score < 18 for detection of dementia and a VCAT cutoff score < 22 for MCI [22]. Lastly, the GDS, with a score range of 0–15 where a higher score reflects more severe depression, was administered to assess for symptoms of depression.

### Effect of language on the VCAT

To study the impact of language difference on the VCAT and MoCA, participants were grouped based on two systems of language classification. The first language classification is the writing system, which includes two subtypes, namely alphabetic and logographic. English, Malay, and Tagalog are examples of the alphabetic subtype, while Mandarin is an example of the logographic subtype. The second system is the language family classification, which includes three subtypes, namely Indo-European, Sino-Tibetian, and Austronesian. English is an example of the Indo-European language, Mandarin belongs to the Sino-Tibetan language, while Malay and Tagalog are examples of the Austronesian languages. Comparison was made among the different language groups within each of the HC and CI groups.

### Statistical analyses

Statistical analyses were performed using SPSS version 21. Descriptives were presented for demographics and cognitive data. Between-group comparisons were performed, where chi-square test was used to compare categorical variables, while Student’s *t* test or Wilcoxon–Mann–Whitney test was used to compare continuous variables. Further analyses using a general linear model (GLM) were performed to adjust for confounding demographic variables while setting diagnosis or language groups as the outcome variable. Diagnostic performance was also measured using the AUC. All statistical tests performed were two-tailed and regarded as significant at *p* < 0.05.

## Results

The total sample of 284 participants consisted of almost the same distribution of males (51.4%) and females (48.6%), mean age 67.93 ± 8.79, and was made up of 52.8% Chinese, 27.1% Malays, 5.6% Indians, and 14.4% Filipinos and other races. The majority of the participants were retirees (66.5%) and the mean years of education was 11.51 ± 3.78.

There were 164 HC and 120 CI participants. Significant group differences were identified in the demographic variable of age (*p* = 0.008) (Table 1). On GLM analyses, after controlling for age, the two groups had significant differences on the MoCA (25.52 ± 3.37 vs 16.59 ± 5.75, *p* < 0.001), total VCAT (22.48 ± 2.50 vs 14.17 ± 5.05, *p* < 0.001) score, and all individual VCAT domain scores (Table 1). In all of the cognitive tests, the HC scored higher than the CI participants. GDS was not significantly different among the two groups.

**Table 1:** Demographic characteristic of HC and CI participants

	HC (*N* = 164)	CI (*N* = 120)	*p* value (univariate)	*p* value (GLM)*
Age				
Mean (SD)	66.88 (8.09)	69.38 (9.51)	0.008	
Years of education				
Mean (SD)	11.60 (3.75)	11.39 (3.84)	0.653	
Gender				
Male (%)	86 (52.4)	60 (50.0)	0.719	
Race, *N* (%)				
Chinese	93 (56.7)	57 (47.5)		
Malay	38 (23.2)	39 (32.5)		
Indian	11 (6.7)	5 (4.2)		
Filipinos and others	22 (13.4)	19 (15.8)	0.220	
Employment, *N* (%)				
Unemployed	23 (14.0)	9 (7.5)		
Employed	36 (22.0)	26 (21.7)		
Retired	104 (63.4)	85 (70.8)	0.203	
Language administered, *N* (%)				
English	63 (38.4)	35 (29.2)		
Mandarin	46 (28.0)	27 (22.5)		
Malay	19 (11.6)	20 (16.7)		
Others	36 (22.0)	38 (31.7)	0.095	
MoCA				
Mean (SD)	25.52 (3.37)	16.59 (5.75)	<0.001	< 0.001
VCAT memory				
Mean (SD)	10.54 (1.83)	5.90 (3.39)	< 0.001	< 0.001
VCAT language				
Mean (SD)	4.07 (0.80)	3.06 (1.13)	< 0.001	< 0.001
VCAT visuospatial				
Mean (SD)	2.28 (0.56)	1.92 (0.74)	< 0.001	< 0.001
VCAT executive function				
Mean (SD)	4.04 (1.66)	2.81 (1.56)	< 0.001	< 0.001
VCAT attention				
Mean (SD)	1.55 (1.35)	0.50 (0.99)	< 0.001	< 0.001
VCAT total				
Mean (SD)	22.48 (3.50)	14.17 (5.05)	< 0.001	< 0.001

*HC* healthy controls, *CI* cognitive impairment, *GLM* general linear model, *SD* standard deviation, *MoCA* Montreal Cognitive Assessment, *VCAT* Visual Cognitive Assessment Test

*GLM adjusted for age

**Fig. 1:**
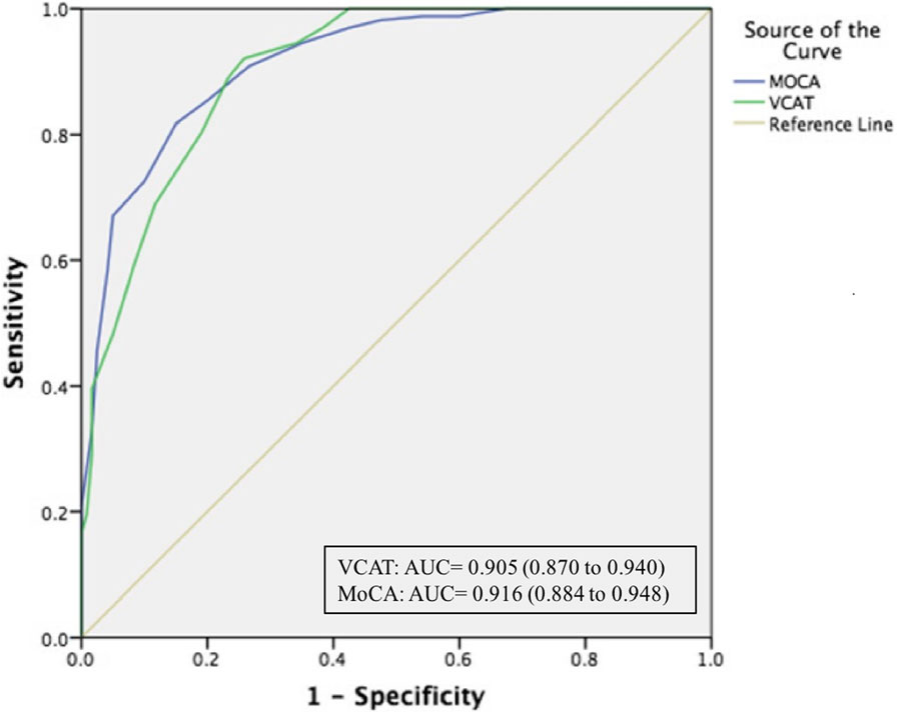
Receiver operating characteristic curves: area under the curve (AUC) for discriminating between HC and CI subjects on VCAT and MoCA scores. *MoCA* Montreal Cognitive Assessment, *VCAT* Visual Cognitive Assessment Test

For discriminating between HC and CI subjects, the AUCs (95% CI) were 0.905 (0.870–0.940) for the total VCAT score and 0.916 (0.884–0.948) for the MoCA score (Fig. 1). For a VCAT cutoff score of 17 which is indicative of cognitive impairment [21], sensitivity is 92.1% and specificity is 74.2%.

### Receiver operating characteristic curves: areas under the curves for discriminating between HC and CI subjects on VCAT and MoCA scores

We studied language differences in the HC group and the CI group independently to remove the influence of disease on the MoCA and VCAT scores. Mean time to complete the VCAT was 10.37 ± 3.70 in the HC group and 13.88 ± 6.18 in the CI group. In the HC, based on the language writing system of classification, 116 HC were placed into the alphabetic group and 48 HC into the logographic group. Mean years of education (*p* < 0.001), gender distribution (*p* = 0.005), and race (*p* < 0.001) were significantly different between the two groups. On GLM analyses which controlled for age, years of education, race, and employment, the logographic group scored significantly higher on the MoCA than the alphabetic group (27.02 ± 2.82 vs 24.91 ± 3.40, *p* = 0.001) but no differences were found for the total VCAT score (22.53 ± 3.53 vs 22.35 ± 3.47, *p* = 0.413) and all individual VCAT domain scores (Table 2). The total VCAT score was comparable between the two groups.

**Table 2:** Demographics, MoCA, and VCAT scores for healthy controls based on language classifications

	Writing system classification					Language family classification			
	Alphabetic (*N* = 116)	Logographic (*N* = 48)	*p* value (univariate)	*p* value (GLM)*	Indo-European (*N* = 63)	Sino-Tibetan (*N* = 46)	Austronesian (*N* = 52)	*p* value (univariate)	*p* value (GLM)**
Age									
Mean (SD)	66.10 (8.39)	68.75 (7.05)	0.520		68.90 (7.39)	68.70 (7.09)	62.79 (8.43)	< 0.001	
Years of education									
Mean (SD)	12.61 (3.69)	9.13 (2.58)	0.000		12.49 (3.33)	9.09 (2.59)	12.88 (4.02)	< 0.001	
Gender									
Male (%)	69 (59.5)	17 (35.4)	0.005		36 (57.1)	16 (34.8)	32 (61.5)	0.642	
Race, *N* (%)									
Chinese	46 (39.7)	47 (97.9)			45 (71.4)	46 (100.0)	0 (0.0)		
Malay	38 (32.8)	0 (0.0)			2 (3.2)	0 (0.0)	36 (69.2)		
Indian	10 (8.6)	1 (2.1)			10 (15.9)	0 (0.0)	0 (0.0)		
Filipinos and others	22 (19.0)	0 (0.0)	0.000		6 (9.5)	0 (0.0)	16 (30.8)	< 0.001	
Employment, *N* (%)									
Unemployed	15 (12.9)	8 (16.7)			7 (11.1)	8 (17.4)	8 (15.4)		
Employed	28 (24.1)	8 (16.7)			3 (4.8)	8 (17.4)	25 (48.1)		
Retired	73 (62.9)	31 (64.6)	0.546		53 (84.1)	29 (63.0)	19 (36.5)	0.001	
MoCA									
Mean (SD)	24.91 (3.40)	27.02 (2.82)	< 0.001	0.001	26.10 (2.76)	27.15 (2.77)	23.38 (3.50)	< 0.001	< 0.001^a,b,c^
VCAT memory									
Mean (SD)	10.41 (1.76)	10.85 (1.98)	0.073	0.130	10.52 (1.67)	10.89 (1.97)	10.33 (1.82)	0.173	0.051^b^
VCAT language									
Mean (SD)	4.10 (0.77)	4.00 (0.85)	0.539	0.909	4.13 (0.77)	4.00 (0.87)	4.10 (0.77)	0.801	0.423
VCAT visuospatial									
Mean (SD)	2.24 (0.57)	2.38 (0.53)	0.188	0.340	2.27 (0.48)	2.39 (0.54)	2.19 (0.66)	0.285	0.480
VCAT executive function									
Mean (SD)	4.22 (1.70)	3.63 (1.48)	0.24	0.710	4.40 (1.65)	3.65 (1.45)	3.96 (1.74)	0.043	0.270
VCAT attention									
Mean (SD)	1.58 (1.35)	1.50 (1.38)	0.704	0.805	1.46 (1.35)	1.57 (1.38)	1.69 (1.34)	0.661	0.709
VCAT total									
Mean (SD)	22.53 (3.53)	22.35 (3.47)	0.794	0.413	22.78 (3.34)	22.50 (3.37)	22.23 (3.79)	0.730	0.071^b^

*MoCA* Montreal Cognitive Assessment, *VCAT* Visual Cognitive Assessment Test, *GLM* general linear model, *SD* standard deviation

*GLM adjusted for years of education, gender, and race

**GLM adjusted for age, years of education, race, and employment

Pairwise comparison between the language family groups (post-hoc contrast test): ^a^difference between Indo-European and Sino-Tibetan; ^b^difference between Sino-Tibetan and Austronesian; ^c^difference between Indo-European and Austronesian

Similar results were also seen when the HC were classified based on language family. On GLM analyses while controlling for age, years of education, race, and employment, MoCA scores (26.10 ± 2.76 vs 27.15 ± 2.77 vs 23.38 ± 3.50, *p* < 0.001) were different among the three groups, with the Sino-Tibetan group scoring highest on the MoCA followed by the Indo-European and then the Austronesian group. However, no significant differences were observed among the groups for the total VCAT score (22.78 ± 3.34 vs 22.50 ± 3.37 vs 22.23 ± 3.79, *p* = 0.073) and its individual domain scores (Table 2).

Among the CI subjects, 90 were classified into the alphabetic group and 30 of them into the logographic group. Years of education (*p* < 0.001), race (*p* < 0.001), and employment status (*p* = 0.012) were significantly different between the two groups. GLM analyses showed that there were no differences between the alphabetic and logographic group on the MoCA (15.98 ± 5.98 vs 18.43 ± 4.61, *p* = 0.306), total VCAT score (14.09 ± 5.27 vs 14.40 ± 4.38, *p* = 0.605), and VCAT individual domain score of memory, language, executive function, and attention (Table 3). However, for the classification based on language family the CI subjects scored significantly different on the MoCA (18.86 ± 4.85 vs 18.48 ± 4.69 vs 14.15 ± 5.94, *p* = 0.002) among the three different groups. The Indo-European and Sino-Tibetan groups were quite similar in their mean MoCA scores and both groups scored better than the Austronesian group. No differences were found for the total VCAT score (14.91 ± 5.54 vs 14.41 ± 4.35 vs 13.56 ± 5.07, *p* = 0.275) and all of its individual domain scores except for visuospatial domain scores (Table 3).

**Table 3:** Demographics, MoCA, and VCAT scores for cognitively impaired participants based on language classifications

	Writing system classification								
	Alphabetic (*N* = 90)	Logographic (*N* = 30)	*p* value (univariate)	*p* value (GLM)*	Indo-European (*N* = 35)	Sino-Tibetan (*N* = 27)	Austronesian (*N* = 55)	*p* value (univariate)	*p* value (GLM)**
Age									
Mean (SD)	68.78 (9.54)	71.17 (9.37)	0.225		73.69 (7.90)	70.26 (9.36)	65.65 (9.22)	< 0.001	
Years of education									
Mean (SD)	12.06 (3.49)	9.47 (4.22)	0.000		11.82 (3.23)	9.26 (4.26)	12.21 (3.67)	0.001	
Gender									
Male (%)	45 (50.0)	15 (50.0)	0.583		16 (45.7)	14 (51.9)	29 (52.7)	0.852	
Race, *N* (%)									
Chinese	27 (30.0)	30 (100.0)			27 (77.1)	27 (100.0)	0 (0.0)		
Malay	39 (43.3)	0 (0.0)			1 (2.9)	0 (0.0)	38 (69.1)		
Indian	5 (5.6)	0 (0.0)			5 (14.3)	0 (0.0)	0 (0.0)		
Filipinos and others	19 (21.1)	0 (0.0)	0.000		2 (5.7)	0 (0.0)	17 (30.9)	< 0.001	
Employment, *N* (%)									
Unemployed	5 (5.6)	4 (13.3)			2 (5.7)	4 (14.8)	3 (5.5)		
Employed	25 (27.8)	1 (3.3)			3 (8.6)	1 (3.7)	22 (40.0)		
Retired	60 (66.7)	25 (83.3)	0.012		30 (85.7)	22 (81.5)	30 (54.5)	0.001	
MoCA									
Mean (SD)	15.98 (5.98)	18.43 (4.61)	0.064	0.306	18.86 (4.85)	18.48 (4.69)	14.15 (5.94)	< 0.001	0.002^a,b,c^
VCAT memory									
Mean (SD)	5.96 (3.39)	5.73 (3.46)	0.738	0.927	6.11 (3.39)	5.63 (3.42)	5.85 (3.41)	0.823	0.756
VCAT language									
Mean (SD)	3.06 (1.12)	3.07 (1.20)	0.914	0.395	3.17 (0.99)	3.04 (1.22)	2.98 (1.19)	0.863	0.210
VCAT visuospatial									
Mean (SD)	1.82 (0.80)	2.20 (0.41)	0.022	0.004	1.77 (0.84)	2.22 (0.42)	1.85 (0.78)	0.059	0.006^a^
VCAT executive function									
Mean (SD)	2.74 (1.62)	3.00 (1.37)	0.315	0.476	3.14 (1.75)	3.07 (1.41)	2.49 (1.49)	0.091	0.099
VCAT attention									
Mean (SD)	0.53 (1.03)	0.40 (0.86)	0.652	0.198	0.71 (1.27)	0.44 (0.89)	0.42 (0.83)	0.488	0.369
VCAT total									
Mean (SD)	14.09 (5.27)	14.40 (4.38)	0.764	0.605	14.91 (5.54)	14.41 (4.35)	13.56 (5.07)	0.005	0.275

*MoCA* Montreal Cognitive Assessment, *VCAT* Visual Cognitive Assessment Test, *GLM* general linear model, *SD* standard deviation

*GLM adjusted for years of education, race, and employment

**GLM adjusted for age, years of education, race, and employment

Pairwise comparison between the language family groups (post-hoc contrast test): ^a^difference between Indo-European and Sino-Tibetan; ^b^difference between Sino-Tibetan and Austronesian; ^c^difference between Indo-European and Austronesian

## Discussion

This study investigated the performance of the VCAT in a multinational, multilingual Southeast Asian cohort across HC and CI participants. The overall discriminative ability was comparable between the VCAT and the MoCA where the AUCs for MoCA and total VCAT were similar in discriminating between HC vs CI participants. The effect of language differences on the VCAT and MoCA was also explored in the study, where participants were classified into groups based on both writing system and language family. In contrast to the distinct language differences in responses to MoCA, a lack of between-group differences that were observed on the VCAT scores suggests that the VCAT is less likely to be influenced by language of administration, and hence will not require translation to the multiple languages spoken across Southeast Asia.

As shown in our previous study and in this present study, as a cognitive screening tool the VCAT shows satisfactory discriminative validity in differentiating between CI participants from HCs. A low score on the VCAT helps clinicians to accurately identify patients with cognitive impairment who would require further investigations. Therefore, the VCAT may improve the early identification and detection of cognitive impairment.

Based on the language classification, despite using translated and validated versions of the MoCA in different language groups, it appears that the language of test administration continues to influence the MoCA scores. Thus a participant scoring 21 on the English MoCA might not necessarily be experiencing the same level of cognitive impairment as a participant who scored 21 on the Malay or Tagalog MoCA. Previous studies investigating the modified MMSE also showed such differences in performance between English and French speakers and observed that the rates of diagnosis for dementia were different between the two language groups [30, 31]. This could be attributable to the translation and adaptation process of the original test version, which gave rise to test items that varied in difficulty, discrimination, and psychometric properties across different language groups [32]. This large variability in test performance poses a challenge for clinicians and researchers across Southeast Asia. Having differing test scores for well-defined cognitive disorders would result in clinicians being unable to compare treatment responses across Southeast Asian countries. From a research perspective, correlation of cognitive performance to biomarker studies and performing multicenter clinical trials with cognitive outcomes would therefore be not feasible in Southeast Asia.

On the contrary, the VCAT did not show any significant group differences in both the writing system and language family classification group comparisons. The VCAT scores were stable between the logographic and alphabet group and among the Indo-European, Sino-Tibetian, and Austronesian groups. This could be attributable to the fact that the VCAT does not require any translation and furthermore the test items were constructed such that the language of administration does not influence test item scores. As such, all of the test items were kept constant regardless of the language administered. This allowed for the standardization of test difficulty and retention of its original intended neuropsychological constructs. The total VCAT score and domains of memory, language, executive function, and attention were not significantly different between language classification groups in both HC and CI subjects. However, visuospatial scores, while not significantly different among language groups in the HC group, there was a significant difference among CI subjects. While the reason for this is not entirely clear, one explanation is that instructions to evaluate visuospatial domain require lengthy explanation and hence among CI subjects, where there may be some impairment in language ability, language differences continue to impact visuospatial test scores.

Other than the VCAT, there have been several tests, including the Eurotest, Phototest, and Memory Alteration Test, designed to overcome the influence of education and culture in screening for dementia [33–35]. The Eurotest was designed to assess one’s cognition by evaluation of subject’s ability to handle money, and has been demonstrated to be effective for subjects who have low levels of education and those who are illiterate. Likewise, the Phototest, which is a short and simple paradigm that employs identification of pictures, has also been shown to be useful for detecting cognitive impairment among subjects who are illiterate.

The strength of this study is the inclusion of participants from four different countries, allowing for the investigation of the effect of factors such as race, language, and cultural background on the accuracy of the VCAT. However, limitations in this study should also be acknowledged. Firstly, this study only included participants from Asian countries and has a relatively small sample size. As such, the results may not be generalizable to non-Asian populations. Future studies should include a larger sample size, longitudinal cohorts, and non-Asian populations. Furthermore, only the MoCA was included as a comparison in a parallel evaluation to investigate the diagnostic ability of the VCAT. To further assess the performance and reliability of the individual domains of the VCAT, future research should include other domain-specific neuropsychological assessments in a multilingual population. As the subjects in this study had a minimum education of 6 years, the usefulness of the VCAT in cohorts with lower education levels warrants further evaluation. However, by comparing the performance of the MoCA to the VCAT, we showed importantly that whereas the MoCA is language dependent, the VCAT is not. We also acknowledge the lack of inter-rater reliability and test–retest reliability data of the VCAT in non-English languages, which we hope to address in the near future. While we demonstrated that the VCAT is useful for the diagnosis of cognitive impairment of the AD type, the inherent language-free nature of the test items together with the visual nature of the test would render the VCAT less useful for the diagnosis of frontotemporal dementia or AD of the posterior cortical atrophy variant, or for patients with significant visual impairment.

## Conclusions

This study demonstrates that the VCAT is useful in a multinational, multiethnic and multilingual Southeast Asian population. The test will be an effective tool in helping clinicians differentiate HC and CI patients, with lower scores on the VCAT being associated with more severe cognitive impairment. Furthermore, in regions where populations are culturally and linguistically diverse, such as in Southeast Asia, screening tests such as the VCAT transcend language differences and hence will be an effective tool that encourages accurate detection of cognitive impairment. Similarly, international clinical trials which involve participants from different language-speaking nations would find a single standardized version of the VCAT to be useful in making meaningful cross-cultural comparisons. Having a standardized tool that can be used uniformly across different populations to guide diagnosis can also potentially aid epidemiological studies to represent the distribution and determinants of worldwide dementia prevalence more precisely.

## Abbreviations

AD: Alzheimer’s disease
AUC: Area under the curve
CDR: Clinical Dementia Rating
CI: Cognitive impairment
GDS: Geriatric Depression Scale
GLM: General linear model
HC: Healthy control
MCI: Mild cognitive impairment
MMSE: Mini Mental State Examination
MoCA: Montreal Cognitive Assessment
VCAT: Visual Cognitive Assessment Test
